# Test for Cod-Liver Oil

**Published:** 1848-11

**Authors:** C. Hockin


*Test for Cod-Liver Oil.* By C. Hockin.—Among the substances proposed for testing the purity of cod-liver oil, I believe that it is not generally known that strong sulphuric acid is that on which reliance can be best placed. Mixing together, on a porcelain slab, four parts of genuine cod-liver oil and one part of strong sulphuric acid, and stirring with a glass rod, a beautiful and rich violet colour, similar to that of the fumes of iodine, is produced, which, in a few seconds, passes gradually into a dirty brown, the altered portion of the oil separating in irregularly shaped patches from that out of reach of the acid. This remarkable characteristic is not possessed by either olive, almond, seal, whale, or fine sperm oils, nor do I believe by any other fat oil. The reaction varies the appearance from a delicate fawn to dark caramel. The latter is produced with several samples of very light cod-liver oil which are found in the market—a circumstance that induces me to think they have been bleached with chromic acid, or other powerfully deoxidising agent, thus decomposing all the gelatinous matter so abundant in cod-liver oil.—*Ibid.*

